# Metrology for Agriculture and Forestry 2019

**DOI:** 10.3390/s20123498

**Published:** 2020-06-21

**Authors:** Giovanni Battista Chirico, Francesco Bonavolontà

**Affiliations:** 1 Department of Agricultural Sciences, University of Naples Federico II, 80055 Portici (NA), Italy; 2 Department of Electrical Engineering and Information Technology, University of Naples Federico II, 80138 Napoli, Italy; francesco.bonavolonta@unina.it

**Keywords:** precision agriculture, precision forestry, precision farming, smart agriculture, wireless sensors, remote sensing, multispectral imagery, UAV, ICT, IoT

## Abstract

This Special Issue is focused on recent advances in integrated monitoring and modelling technologies for agriculture and forestry. The selected contributions cover a wide range of topics, including wireless field sensing systems, satellite and UAV remote sensing, ICT and IoT applications for smart farming.

## 1. Introduction

Agriculture and forestry must address several issues in the coming decades, from both social and environmental perspectives. Population growth, dietary and lifestyle changes, combined with the increasing global competition in world markets, must be mediated with the need to ensure the environmental sustainability of the production processes, while preserving ecosystems and natural resources [1,2].

Recent advances in monitoring, modelling and communication systems have opened new opportunities for coping with these issues. The research sector has been developing several technologies, often recalled with the terms of “precision” or “smart” agriculture and forestry, that rely on the combination of advanced (both proximal and remote) sensors, automation and control systems, satellite navigation and positioning technology, system modelling, Information and Communication Technologies (ICT) and the Internet of Things (IoT) [3,4,5,6].

These technologies are rapidly growing, with strong investments from the industrial sector, which is offering an increasing number of tools and services. Successful examples already exist, such as operational advisory services based on crop monitoring and real-time data processing [7,8,9]. 

These tools and services are designed to enhance the competitiveness of farm holdings and the overall agri-food chains, by implementing production processes that allow one to produce more with less. This aspect is particularly relevant for the farm holdings of EU Mediterranean countries, which need to compete with the more developed and integrated markets of Northern Europe. They can also enhance the development of business in the agri-food chains, by offering many opportunities to the service industries (e.g., sensor producers, ICT and IOT companies). They also offer opportunities to farmers, by posing a more efficient integration with the industries and services involved into food processing, logistics and retail.

In order to transfer the recent technological innovations into efficient solutions for the agricultural and forestry sectors, a close collaboration is required between researchers, technological manufacturers and farmers. 

For this purpose, the 2019 IEEE International Workshop on Metrology for Agriculture and Forestry was organized at the Department of Agricultural Sciences of the University of Naples Federico II, in Portici (Italy). This special issue collects some selected papers presented during the workshop.

## 2. Contributions

Following a rigorous review process, eleven outstanding papers have been finally selected, covering a wide spectrum of research topics in the broader areas of the Special Issue.

The application of wireless sensing technology was one of the focus topics. A new agro-sensor named AgriLogger was presented [10]. AgriLogger was designed for collecting and transmit temperature and relative humidity in remote areas that cannot be easily reached, and are not served by telecommunication networks. The device can be remotely placed on preselected sites through a customized drone, and it is characterized by low energy consumption, thus ensuring a long-term battery life.

Collecting field agrometeorological data is fundamental for a proper management of agronomic practices. Numerical weather predictions have often been used as a surrogate of field weather data [11,12], however their reliability can be limited if not combined with some field observations [13]. Moreover, the access to full sets of high-resolution weather forecasts can be quite expensive. One paper of this Special Issue [14] proposed a methodology for forecasting crop evapotranspiration, by combining ground weather sensors and air temperature forecasts, which are generally provided with lead times of several days, at no subscription costs, and with high accuracy. The proposed methodology also integrates crop parameters retrieved by multispectral satellite images.

Remote sensing technology has been the topic of another three papers of this Special Issue. Paper [15] presented an approach that combines Sentinel 1 and Sentinel 2 data for land cover mapping, to overcome the limitation of the methods based on Sentinel 2 data, that are unusable in presence of cloud covers. 

The availability of free satellite-based imagery is playing a fundamental role in promoting precision agricultural technologies, especially for crop parameter mapping, crop status monitoring and disease assessment. However, the resolution of free satellite images is too low when crops are grown by rows. Data retrieved from satellite imagery could be biased by intra-row covering, giving inaccurate information about crop status. Paper [16] presented a novel procedure, designed to improve the satellite imagery. The proposed procedure exploits high resolution images acquired by airborne multispectral sensors on unmanned aerial vehicle (UAV). Satellites images are refined by assimilating UAV multispectral data with a convolutional neural network. UAV multispectral images were also applied in [17] for investigating the impacts of long-term conservation agricultural practices on crop yield as well as on vegetation indices. The results showed that soil management affects the vegetation indices accuracy, which is related to the nitrogen nutrition status. 

Advanced sensing technologies are also used in controlled environments in combination with crop growth models. Paper [18] applied this technology for predicting crop responses to microclimatic changes, as part of decision support systems designed for optimal crop system management in controlled environments.

An exemplary application of IoT in smart farming is provided by [19]. An IoT platform based on a low-cost, modular and long-range network of sensors was designed for collecting environmental data related to the growth of farm products. 

An integrated application of ICT with system modelling is instead presented by [20]. The system was designed to monitor the areas under fire-risk and to assess the optimal evacuation routes for wild animals in cases of wildfire.

Advanced sensing technologies were also proposed for a precision apiculture system platform, designed to monitor and control the relevant environmental factors affecting honey production [21]. Smart sensors were also proposed for the real-time and long-term measurement of relevant parameters related to beehive conditions, such as the hive weight and sounds emitted by the bees [22]. The fusion of different sensor measurements may provide insights into the status of the colony, its interaction with the surrounding environment, and the influence of climatic conditions. Indeed, as illustrated by [23], smart sensing technology can be accounted for as an evolution of precision farming, and it has its technological basis very close to the paradigms of Industry 4.0, which underlines the need to use Integrated Information Systems (IIS) to manage all activity areas of any production system. 
